# Comparative effectiveness of adalimumab versus infliximab in children with Crohn’s disease: real-world data from the prospective PIBD-SETQuality inception cohort study

**DOI:** 10.1093/crocol/otag069

**Published:** 2026-07-03

**Authors:** Stephanie A Vuijk, Renz C W Klomberg, Eva Katgert, Margreet M S Wessels, Erasmo Miele, Fiona L Cameron, Ivan D Milovanovich, Rafeeq Muhammed, David Devadason, James Hart, Dimitris Rizopoulos, Nicholas M Croft, Lissy de Ridder, the PIBD-SETQuality Consortium, Dan Turner, Gili Focht, Nicholas Croft, Lissy de Ridder, Gigi Veereman, Sibylle Koletzko, Annecarin Brückner, Richard Russell, Dror Weiner, Anne Griffiths, Marina Aloi, Thomas Walters, Frank Ruemmele

**Affiliations:** Department of Pediatric Gastroenterology, Erasmus Medical Centre—Sophia Children’s Hospital, Rotterdam, the Netherlands; Department of Pediatric Gastroenterology, Erasmus Medical Centre—Sophia Children’s Hospital, Rotterdam, the Netherlands; Department of Pediatric Gastroenterology, Erasmus Medical Centre—Sophia Children’s Hospital, Rotterdam, the Netherlands; Department of Pediatrics, Rijnstate Hospital, Arnhem, the Netherlands; Department of Translational Medical Science, Section of Paediatrics, University of Naples ‘Federico II’, Naples, Italy; Department of Pediatric Gastroenterology, Alderhey Children’s Hospital NHS Trust, Liverpool, United Kingdom; Department of Pediatric Gastroenterology, University of Liverpool, Liverpool, United Kingdom; Department of Gastroenterology, Hepatology, and Endoscopy, University Children’s Hospital, Tisova Str. No 10, Belgrade, Serbia; Faculty of Medicine, University of Belgrade, Dr Subotica Str. 8, Belgrade, Serbia; Department of Pediatric Gastroenterology, Birmingham Children’s Hospital, Birmingham, United Kingdom; Department of Pediatric Gastroenterology, Nottingham University Hospitals NHS Trust, Nottingham, United Kingdom; Pediatric Gastroenterology, Royal Devon and Exeter Hospital NHS Trust, Exeter, United Kingdom; Department of Biostatistics, Erasmus MC, Rotterdam, the Netherlands; Department of Epidemiology, Erasmus MC, Rotterdam, the Netherlands; Pediatric Gastroenterology, Centre for Immunobiology, Blizard Institute, Barts and the London School of Medicine, Queen Mary University of London, London, United Kingdom; Department of Pediatric Gastroenterology, Erasmus Medical Centre—Sophia Children’s Hospital, Rotterdam, the Netherlands

**Keywords:** anti-TNF, inflammatory bowel disease, pediatric

## Abstract

**Background:**

Infliximab and adalimumab are effective anti-tumor necrosis factor (anti-TNF) therapies for the treatment of pediatric Crohn’s disease (CD). The aim of this study was to compare the effectiveness of infliximab and adalimumab in a real-world cohort of children with CD.

**Methods:**

Data from biological-naïve children with luminal CD (age 3-18 years) who commenced anti-TNF and completed at least 1 year of follow-up were collected from the prospective multicenter observational PIBD-SETQuality study. The primary outcome was steroid-free clinical remission (SFCR), defined as clinical remission (weighted pediatric Crohn’s disease activity index [wPCDAI] <12.5) without systemic steroids or luminal surgery at 1 year. The relative risk (RR) of SFCR was calculated using standardization, correcting for the following baseline covariates: age, upfront anti-TNF, C-reactive protein, erythrocyte sedimentation rate, albumin, leukocytes, disease behavior, wPCDAI, perianal disease, and concomitant immunomodulator use. Secondary outcomes included the durability of anti-TNF treatment without luminal surgery.

**Results:**

Between January 1, 2017, and June 14, 2024, 178 patients with anti-TNF were included (infliximab: *n* = 121 [68%], adalimumab: *n* = 57 [32%]). At 12 months, 34/56 (61%) patients treated with adalimumab and 66/120 (55%) patients treated with infliximab had reached SFCR. The RR of SFCR at 1 year with adalimumab compared to infliximab was 1.25 (95% confidence interval [CI] 0.94-1.66], *P* = .13. Adalimumab was associated with a significant lower adjusted hazard ratio (aHR) of treatment discontinuation than infliximab in patients with a concomitant immunomodulator (aHR 0.17 [95% CI 0.04-0.75], *P* = .020), adjusted for upfront anti-TNF.

**Conclusions:**

In this prospective cohort of children with CD, adalimumab and infliximab showed comparable clinical effectiveness 1 year after the start of anti-TNF treatment.

**Clinical trial registration:**

The ClincalTrials.gov ID of this study is NCT03571373.

Key Messages *What is already known?*

Previous observational studies showed similar outcomes between infliximab and adalimumab in children with Crohn’s disease, but high-quality prospective, real-world data remains limited.

*What is new here?*

In this unique, international, prospective cohort, it was shown that after 1 year there was no significant difference in steroid free clinical remission between infliximab and adalimumab in 178 children with Crohn’s disease (relative risk of steroid free remission for adalimumab vs infliximab: 1.25 [95% confidence interval 0.94-1.66], *P* = .13).

*How can this study help patient care?*

These results indicate infliximab and adalimumab can be considered similarly effective treatment options, supporting informed treatment choices for patients and physicians in managing pediatric Crohn’s disease.

## Introduction

According to the most recent European ECCO-ESPGHAN guideline for pediatric Crohn’s disease (CD), both infliximab and adalimumab are safe and effective treatment options for induction and maintenance of remission.^1^ Both treatments inhibit the interaction between the inflammatory cytokine tumor necrosis factor-alpha (TNF-α) and its receptor, thereby reducing inflammatory responses of the immune system.^2^ The current standard maintenance regimen of infliximab, a human–mouse recombinant IgG monoclonal antibody approved for pediatric CD since 2006, is intravenous administration every 8 weeks,^1^^,^^3^ while adalimumab has been approved for pediatric CD since 2014 and is an entirely human IgG monoclonal antibody administered subcutaneously every other week, although pediatric patients often require a more intensified dosing.^1^^,^^3^ The ECCO-ESGPHAN guideline recommends starting a concomitant immunomodulator, such as a thiopurine or methotrexate, to prevent anti-drug antibody development when commencing infliximab, while adalimumab may be given as monotherapy.^1^ However, the recommendations for combination treatment may differ between regions.^4^^,^^5^

The choice between infliximab and adalimumab may be influenced by various factors. These can include physicians’ and patients’ preferences, such as route of administration, distance to the hospital, or treatment costs. Additionally, there is a tendency to prescribe infliximab in patients who have higher clinical and endoscopic disease severity or in patients who have perianal disease.^6^^,^^7^ In adults, previous studies comparing infliximab and adalimumab showed no consistent significant differences in effectiveness between the 2 therapies.^8–11^ In children with CD, most observational studies showed no significant differences in clinical outcomes between infliximab and adalimumab, although results from an Italian Registry including pediatric patients with IBD showed favorable durability of adalimumab compared to infliximab.^7^^,^^12–15^ More high-quality prospective, international multicenter data are needed to better understand the real-world effectiveness of infliximab and adalimumab and improve personalized treatment.

The primary aim of this study was to compare the 12-month outcomes of adalimumab and infliximab in a multicenter prospective observational study in children with CD. Based on prior studies, we hypothesized that there would be no difference in clinical effectiveness at 12 months.

## Materials and methods

### Study design and participants

Data were collected from patients recruited between January 1, 2017, and June 14, 2024, from the Pediatric Inflammatory Bowel Diseases (IBD) Network for Safety, Efficacy, Treatment and Quality (PIBD-SETQuality) inception cohort, an international prospective observational study.^16^ Children who were 3-18 years old with a confirmed diagnosis of CD based on the revised Porto criteria, who were biologic-naïve and had an indication to start anti-TNF treatment for luminal disease during follow-up were eligible for inclusion in this analysis.^17^ Patients were allowed to also have perianal disease. The minimum follow-up time was 12 months after the start of anti-TNF.

### Data collection and definition of variables

In the PIBD-SETQ study, visits were scheduled at 0, 4, 12, 26, 52, and 78 weeks after the start of the first induction treatment, followed by yearly visits. For the purpose of this analysis, the start date of anti-TNF treatment was considered as baseline. To account for the possible misalignment of PIBD-SETQ study visits and start dates of anti-TNF treatment, visits up to 12 weeks before the start of anti-TNF and 1 week after the start of anti-TNF were eligible for use of baseline data. For outcome analyses at 12 months after anti-TNF initiation, visits within a timeframe of ±12 weeks were permitted.

At each study visit, the following data were collected: clinical disease activity, as scored by the weighted pediatric CD index (wPCDAI) and grouped into remission, mild, moderate or severe according to validated cutoffs,^18^ or by the physician global assessment (PGA); laboratory results, including C-reactive protein (CRP), erythrocyte sedimentation rate (ESR), albumin, and white blood cell (WBC) count; IBD-related serious adverse events (SAEs), side effects of biological treatment; and therapy details. A concomitant immunomodulator was defined as one that was initiated before or within 2 weeks of starting anti-TNF therapy, and was given for at least 1 week after start of anti-TNF. If a patient switched from subcutaneous methotrexate to oral methotrexate or vice versa, this was considered as a single period of concomitant immunomodulator use. Side effects of treatment were pre-specified as serious infection, abdominal pain, headache, opportunistic infection, arthritis, psoriasiform skin eruption, (delayed) infusion reaction, injection site reaction, or other. Additionally, a fecal calprotectin was requested at specific study visits (at diagnosis, 12 weeks after diagnosis, and yearly after diagnosis), but when fecal calprotectin was measured more frequently, these values were also reported. At diagnosis, the Paris classification was recorded, which was updated yearly.^19^ Results from endoscopy and bowel imaging at diagnosis, and if available, during follow-up were recorded. We used the most recent results from endoscopy and bowel imaging before the start of anti-TNF to classify disease location, disease behavior and Simple Endoscopic Score for Crohn’s Disease (SES-CD) score. Study data were collected and managed using REDCap electronic data capture tools hosted at Queen Mary University of London.^20^

### Outcome measures

The primary outcome was steroid-free clinical remission (SFCR) at 12 months after the start of anti-TNF treatment, defined as clinical remission (a wPCDAI <12.5 or if unavailable, a PGA indicating remission), without luminal surgery (perianal surgery was allowed) and without additional systemic corticosteroid use within 12 months after start of anti-TNF treatment. Additional systemic corticosteroid use was defined as systemic corticosteroids started >1 week after starting anti-TNF, or systemic corticosteroids started before anti-TNF initiation but continued for >8 weeks after start of anti-TNF therapy. If a patient switched from one type of corticosteroids to another, the start date of the first corticosteroid was taken into account. IV corticosteroids administered on the day of infliximab infusion to prevent an infusion reaction were not included in this definition. Secondary dichotomous outcomes included steroid-free mild or inactive disease (SFMI; defined using the same criteria as for SFCR but allowing for both mild disease or remission), normal CRP remission (NCR: CRP <5 mg/L and clinical remission without luminal surgery), and normal fecal calprotectin remission (NFR: fecal calprotectin <250 μg/g and clinical remission without luminal surgery) at 12 months after start of anti-TNF treatment. Patients who discontinued anti-TNF treatment before 12 months due to primary non-response/inadequate response (PNR), loss of response (LOR), development of antibodies, non-adherence, or side effects were considered to have not achieved these primary or secondary outcomes; those who discontinued anti-TNF before 12 months as per physician’s discretion due to sustained remission were still eligible to have achieved the outcome. Furthermore, the proportion of patients with ≥1 SAE and ≥1 side effect of treatment at 12 months was assessed. IBD-related SAEs included luminal surgery or any AE that was life threatening or led to (prolonged) hospitalization. Last, we evaluated anti-TNF durability, defined as the time on anti-TNF since the initiation of anti-TNF treatment to anti-TNF cessation or luminal surgery, censored at last follow-up date. Patients who discontinued anti-TNF due to sustained remission were considered not to have experienced the event, and the date of anti-TNF withdrawal was used as their censoring date.

### Statistical analyses

Baseline characteristics were analyzed using descriptive statistics. Numerical data were reported as mean with standard deviation if normally distributed or median and interquartile range if not normally distributed. Categorical data were presented as frequencies and proportions. To compare continuous variables, independent sample *t*-tests or Mann–Whitney *U* tests were used, while categorical variables were compared with the Chi-square or Fisher’s Exact tests, as appropriate. A sample size was not calculated as it is not meaningful to calculate a sample size retrospectively.

Relative risks (RRs) were calculated for dichotomous outcomes. Since PIBD-SETQuality is an observational study, the treatment assignment to either infliximab or adalimumab was not randomized so RRs cannot be directly interpreted causally. We used the method of standardization combined with penalized logistic regression to obtain estimates of the RRs of achieving the outcome with adalimumab versus infliximab. This approach involves developing a model to predict the outcome based on key confounders that could potentially influence the outcome and the choice of treatment.^21^^,^^22^ We constructed a penalized logistic regression model to predict the expected outcome for each participant under both treatments, while accounting for these confounders. Hence, the results can be interpreted as crude RRs. Covariates were pre-selected considering previous literature and the possible association with both the outcome measurements and choice of treatment.^1^^,^^12^^,^^23–25^ To account for a larger number of confounders while avoiding overfitting, we fitted a penalized logistic regression model and applied principal component analysis (PCA) to most numerical confounders. PCA is a dimensionality reduction technique that transforms the original variables into a smaller set of uncorrelated components, capturing most of the variability in the data. This way, more numerical confounders could be included. In addition to anti-TNF type (adalimumab vs infliximab), 7 other confounders could be included. These included the first 2 principal components based on several numerical variables (CRP, ESR, albumin, WBC count, age at start anti-TNF treatment in years, and time from CD diagnosis to anti-TNF treatment in weeks), wPCDAI, and categorical variables, including disease behavior (B2 and/or B3 vs B1), disease location (L3 vs L2 vs L1), perianal disease and concomitant immunomodulator use. One patient classified as isolated upper GI disease (L4) only had extensive jejunal and proximal ileal inflammation (L4b). For the purpose of the analysis, the patient was classified as L1 disease. When wPCDAI at start of anti-TNF treatment was unavailable (*n* = 48), this value was imputed based on PGA using the midpoint of the wPCDAI range: 6.25 for remission, 26.25 for mild, 48.75 for moderate, and 91.25 for severe.^18^ Fecal calprotectin and SES-CD scores were not included as these covariates had too many missing values, and the SES-CD score could also be from a endoscopy performed at diagnosis and therefore not reflect the (unmeasured) SES-CD score around start of anti-TNF.

We used a multivariable Cox proportional hazards (PHs) model to evaluate the durability of anti-TNF treatment. Variables included in this model were anti-TNF type (adalimumab vs infliximab), timing of anti-TNF initiation (upfront vs no upfront therapy), and concomitant immunomodulator use. Additionally, we included the interaction term between anti-TNF type and concomitant immunomodulator. Even though the model fit did not improve when including this interaction term based on the Akaike Information Criterion/Bayesian Information Criterion and likelihood ratio test (results not shown), it was decided to keep this interaction term, as studies have shown conflicting results regarding the benefit of immunomodulators with infliximab and adalimumab. Due to the small number of events covariates reflective of disease severity at baseline (such as wPCDAI and CRP) could not be included. The PH assumption were tested using the Schoenfeld residuals. The PH assumption was more questionable for the covariate concomitant immunomodulator (Figure S1). Due to the small number of events, it was decided not to take any further action to correct this. Cumulative survival probabilities at 1, 2, and 3 years were calculated from the Cox model using the Breslow estimator. A Kaplan–Meier analysis was performed to obtain the unadjusted survival probabilities for adalimumab and infliximab at 1 year.

### Missing values

The outcome SFCR and SFMI had missing data for 2/178 (1%) of patients, NCR had missing data for 10/178 (6%) patients, and FCR had missing data for 53/178 (30%) of patients (which was mostly due to unavailable calprotectin data at 12 months which were missing in 92/178 [52%] patients). Outcome data were not imputed. The percentage of missing values across the 12 covariates included in our regression model for standardization varied between 0.6 and 12% (disease location 0.6%, CRP and albumin both 5%, WBC count 8%, ESR 12%, other covariates no missing values). For analysis of our primary outcome, 36 out of 176 (20%) cases had incomplete data on any or more of these covariates. To improve accuracy and statistical power, we used multiple imputation to create and analyze 30 imputed datasets. Incomplete variables were imputed under fully conditional specification, using the default settings of the mice package. All variables included in the regression model were used as predictor in the imputation model. Missing data were internally validated by simulating missingness in 5% of the observed data and comparing the imputed values with the original values to assess the accuracy of the imputation process. Outcomes of interest were estimated in each imputed dataset separately and combined using Rubin’s rules. As a sensitivity analysis, we also performed the analysis for the primary outcome on the subset of complete cases.

It was anticipated that there were many missing values for fecal calprotectin, as it was not part of the study protocol to obtain fecal calprotectin values at each study visit. Because fecal calprotectin was missing in >50% of cases, it was decided not to impute these data. Patients with missing fecal calprotectin values at 12 months were less likely to have moderate or severe disease, though this was borderline not significant (*P* = .057) (Table S1). Therefore, NFR was reported as an exploratory outcome after concluding that the missingness pattern was not completely missing at random. RRs were not calculated for exploratory outcomes. All analyses were performed using R Statistical Software (version 4.3.3; R development Core Team 2021, Vienna). R packages which have been used for analysis have been cited in Supplementary Material.

### Ethical statement

Medical ethical approval for the PIBD-SETQuality study was previously granted by the ethics committee of each participating medical center. Informed consent was obtained from all patients and, if required by national regulations, their parents.

## Results

### Study population

**Figure 1:**
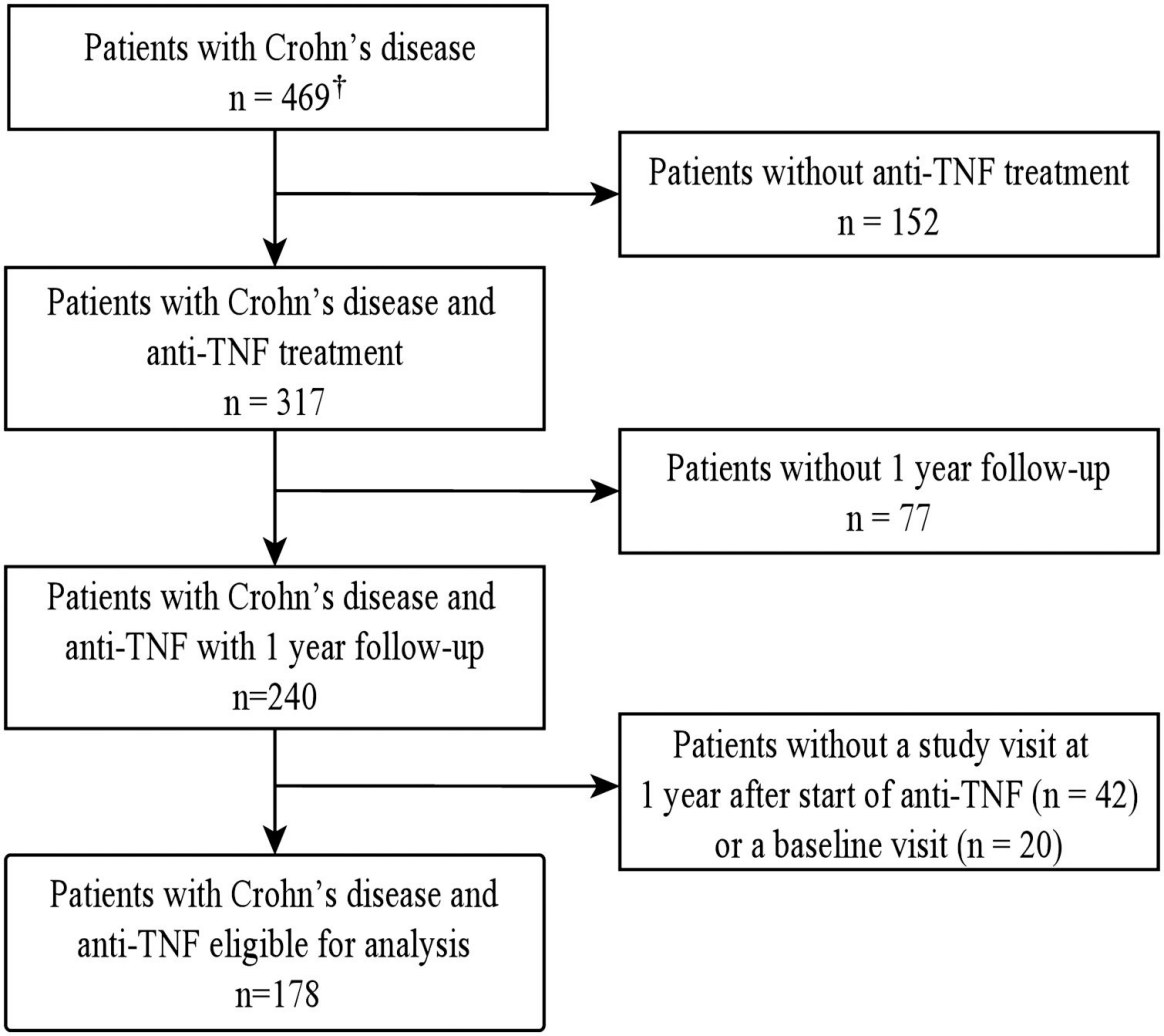
Flowchart of patient selection of patients with Crohn’s disease who initiated anti-TNF treatment. ^†^Seven patients were also included in the TISKids study.^26^ These patients started with 5 infusions of infliximab after which infliximab therapy was discontinued per protocol and azathioprine monotherapy was continued. These patients were excluded from this analysis. TNF, tumor necrosis factor.

From January 1, 2017, until June 14, 2024, a total of 469 patients diagnosed with CD or IBD-unclassified favoring CD, were recruited (Figure 1). Patients who never received anti-TNF treatment were excluded (*n* = 152). Seventy-seven patients were excluded due to insufficient follow-up (no follow-up information available 1 year after the start of anti-TNF treatment). Of the 240 patients with anti-TNF and follow-up longer than 1 year, 178 (74%) patients had a study visit available at 12 months after start of anti-TNF therapy (with a margin of 12 weeks) and a baseline visit (with a margin of 12 weeks before to 1 week after start of anti-TNF). A total of 121 (68%) patients were treated with infliximab, and 57 (32%) patients were treated with adalimumab as first biological.

### Baseline characteristics

Baseline characteristics at start of anti-TNF treatment are depicted in Table 1. The median time since diagnosis to anti-TNF treatment was 5.0 (interquartile range [IQR] 2.0-17.0) weeks for infliximab and 8.0 (IQR 3.0-17.0) weeks for adalimumab (*P* = .223) (Table 1). The choice for either infliximab or adalimumab varied significantly between countries. For instance, out of all patients receiving anti-TNF in the Netherlands (*n* = 67), 62 (93%) received infliximab and only 5 (7%) received adalimumab; in the United Kingdom, out of all patients receiving anti-TNF (*n* = 71), 30 (42%) received adalimumab and 41 (58%) received infliximab; in Israel, 10/11 (91%) received adalimumab and 1/11 (9%) received infliximab. A significantly higher proportion of patients receiving infliximab started their anti-TNF therapy as upfront treatment (meaning anti-TNF was the initial induction therapy after diagnosis). Seventy-five percent (91/121) of patients treated with infliximab and 60% (34/57) of patients treated with adalimumab received a concomitant immunomodulator (*P* = .052). Duration of concomitant immunomodulator therapy from the start of anti-TNF treatment until either the stop of concomitant immunomodulator therapy or the visit at 1-year follow-up (whichever came first) for infliximab-treated patients was 46 weeks (IQR 34-53 weeks), and for adalimumab-treated patients was 45 weeks (IQR 34-53 weeks). Of patients who had a concomitant immunomodulator, 85 (97%) infliximab and 27 (87%) adalimumab treated patients still used an immunomodulator at 12 weeks, and 73 (83%) infliximab and 26 (84%) adalimumab treated patients still used an immunomodulator at 26 weeks. Clinical disease activity, CRP and ESR were significantly higher, whereas albumin levels were significantly lower, in patients receiving infliximab compared to those receiving adalimumab, reflecting more severe disease. Among patients receiving infliximab, the proportion of patients with perianal disease was numerically higher than among those receiving adalimumab, but this was not significant (34% vs 21%, *P* = .116).

**Table 1:** Patient and disease characteristics of children with Crohn’s disease at start of anti-TNF treatment.

	IFX (*n* = 121, 68%)	ADA (*n* = 57, 32%)	*P*-value
**Age at anti-TNF initiation (years), median (IQR)**	13.9 (11.5-15.7)	13.6 (11.9-15.1)	.669
**Male sex, *n* (%)**	78 (64)	36 (63)	.999
**Country, *n* (%)**			**<.001**
** The Netherlands**	62 (51)	5 (9)	
** United Kingdom**	41 (34)	30 (53)	
** Italy**	4 (3)	2 (4)	
** Serbia**	4 (3)	5 (9)	
** Germany**	3 (2)	2 (4)	
** France**	0 (0)	1 (2)	
** Israel**	1 (1)	10 (18)	
** Malaysia**	2 (2)	0 (0)	
** Japan**	4 (3)	2 (4)	
**Time between diagnosis and treatment initiation (weeks), median (IQR)**	5.0 (2.0-17.0)	8.0 (3.0-17.0)	.223
**Upfront anti-TNF treatment, *n* (%)^a^**	53 (44)	12 (21)	**.006**
**Paris classification, *n* (%)**			
** Age at diagnosis**			.945
** A1a (<10 years)**	19 (16)	10 (18)	
** A1b (10-<17 years)**	97 (80)	45 (79)	
** A2 (17-40 years)**	5 (4)	2 (4)	
** Location^b^**			
** L1-L4 subcategories**			.270
** L1 (terminal ileal ± limited cecal disease)**	22 (18)	15 (26)	
** L2 (colonic)**	22 (18)	10 (18)	
** L3 (ileocolonic)**	76 (63)	31 (54)	
** L4 (isolated upper disease)**	0 (0)	1 (2)	
** L4 subcategories^b^**			.375
** L4a (upper disease, proximal to ligament of Treitz)**	64 (53)	23 (40)	
** L4b (upper disease, distal to ligament of Treitz and proximal to distal 1/3 ileum)**	9 (7)	4 (7)	
** L4ab (both L4a and L4b)**	1 (1)	0 (0)	
** No upper disease**	47 (39)	29 (52)	
** Behavior**			.168
** B1 (non-stricturing, non-penetrating)**	98 (81)	48 (84)	
** B2 (stricturing disease)**	10 (8)	8 (14)	
** B3 (penetrating disease)**	9 (7)	1 (2)	
** B2B3 (stricturing and penetrating disease)**	4 (3)	0 (0)	
** Perianal disease**	41 (34)	12 (21)	.116
**Weight (kg), mean (SD)^b^**	43.0 (15.0)	44.7 (14.8)	.501
**Weight z-score, median (IQR)^b^**	−0.74 (−1.74 to 0.20)	−0.50 (−1.25 to 0.29)	.228
**Height (cm), median (IQR)^b^**	159 (145-170)	157 (148-172)	.901
**Height z-score, mean (SD)^b^**	−0.09 (1.27)	−0.32 (1.37)	.483
**BMI (kg/m^2^), median (IQR)^b^**	16.8 (14.8-18.6)	16.5 (15.6-18.4)	.647
**BMI z-score, median (IQR)^b^**	−1.09 (−2.13 to −0.06)	−1.06 (−1.68 to −0.32)	.606
**wPCDAI, median (IQR)^b^**	42.5 (17.5-60.0)	17.50 (7.5-27.5)	**<.001**
**Clinical disease activity, *n* (%)**			**.009**
** Remission**	20 (17)	17 (30)	
** Mild**	37 (31)	25 (44)	
** Moderate**	32 (26)	7 (12)	
** Severe**	32 (26)	8 (14)	
**Laboratory results, median (IQR)^b^**			
** CRP, mg/L**	16 (6-39)	7 (3-20)	**.009**
** ESR, mm/hr**	30 (16-48)	20 (10-35)	**.004**
** Albumin, g/L**	37 (32-42)	40 (34-42)	**.041**
** WBC count, 10^9^/L**	9 (7-11)	8 (6-10)	.051
** Fecal calprotectin, µg/g**	1707 (807-2847)	1155 (474-1857)	.105
**Latest SES-CD score before start anti-TNF^b^**	15 [10-20]	12 [7-18]	.227
** Time endoscopy to start anti-TNF (weeks)**	5 [2-14]	9 [3-16]	.199
**Previous luminal surgery, *n* (%)**	3 (2)	0 (0)	.552
**Previous therapies, *n* (%)**			
** Immunomodulators^c^**	8 (7)	10 (18)	**.047**
** Thiopurines**	8 (7)	7 (12)	
** Methotrexate**	1 (1)	4 (7)	
** Corticosteroids**	19 (16)	12 (21)	.505
**Concomitant therapies, *n* (%)**			
** Immunomodulators**	91 (75)	34 (60)	.052
** Thiopurines**	83 (69)	26 (46)	
** Methotrexate**	8 (7)	8 (14)	
** Corticosteroids**	21 (17)	7 (12)	.518
** Time on concomitant immunomodulator therapy (weeks), median (IQR)^b^**	46 (34-53)	45 (34-53)	.757

Abbreviations: ADA, adalimumab; BMI, body mass index; CRP, C-reactive protein; ESR, erythrocyte sedimentation rate; IFX, infliximab; SES-CD, simple endoscopic score for Crohn’s disease; IQR, interquartile range; TNF, tumor necrosis factor; WBC, white blood cell. P-values <0.05 have been made bold.

a Anti-TNF was the intended first induction therapy. *Z*-scores were calculated based on growth reference standards from the United States Center for Disease Control and Prevention (CDC).

b The following variables had missing values (*n* = number of missing values): disease location (*n* = 1), upper disease location (*n* = 1), weight (*n* = 4), weight *z*-score (*n* = 5), height (*n* = 86), height *z*-score (*n* = 87), BMI (*n* = 86), BMI *z*-score (*n* = 87), wPCDAI (*n* = 48),CRP (*n* = 8), ESR (*n* = 21), Albumin (*n* = 9), WBC (*n* = 14), fecal calprotectin (*n* = 89), SES-CD score (*n* = 55), time endoscopy to start anti-TNF (*n* = 11), time on concomitant immunomodulator therapy (*n* = 6).

c Patients might have used more than 1 immunomodulator.

### Primary outcome

The median time from start of anti-TNF until the 12-month visit was 52 weeks (IQR 47-55) for infliximab-treated patients and 50 weeks (IQR 47-54) weeks for adalimumab-treated patients (*P* = .456). At 12 months, 34/56 (61%) patients treated with adalimumab and 66/120 (55%) patients treated with infliximab had reached SFCR. Of those patients who did achieve the primary outcome, 4 patients had withdrawn anti-TNF (all infliximab-treated patients) due to sustained clinical remission and were still in remission while on immunomodulator use. The crude RR of achieving SFCR at 12 months with adalimumab compared to infliximab was 1.25 (95% confidence interval [CI], 0.94-1.66, *P* = .13). The sensitivity analysis using the complete cases showed a smaller magnitude of difference between the 2 treatments, with a crude RR of 1.11 (95% CI, 0.79-1.56, *P* = .53). This result is based on fewer patients (*n* = 140) and makes the strong assumption that the missing data are completely at random.

### Secondary outcomes

#### Steroid-free mild/inactive disease, normal CRP remission, and normal fecal calprotectin remission

At 12 months after anti-TNF initiation, 45/56 (80%) reached SFMI with adalimumab and 90/120 (75%) with infliximab (crude RR 1.14 [95% CI, 0.94-1.38], *P* = .18). At 12 months after anti-TNF initiation, 30/51 (59%) patients treated with adalimumab and 62/117 (53%) patients treated with infliximab reached NCR (crude RR 1.20 [95% CI, 0.91-1.58], *P* = .19). Based on available fecal calprotectin data, there was no difference in the proportion of patients in NFR between patients treated with infliximab (*n* = 34/92 [37%]) or adalimumab (*n* = 9/33 [27%], *P* = .43).

#### Adverse events

In total, 18/121 patients (10%) had 1 or more SAEs within 12 months after start of anti-TNF treatment (Table 2). There was no significant difference in the proportion of SAEs between patients treated with infliximab (*n* = 16/121, 13%), and those treated with adalimumab (*n* = 2/57, 4%), *P* = .08. Within 12 months, there were no differences in the number of registered side effects between infliximab (*n* = 30/121, 25%) and adalimumab (*n* = 7/57, 12%) (Table S2).

**Table 2:** Serious adverse events within 12 months after start of anti-TNF treatment in children with Crohn’s disease.

	IFX (*n* = 121)	ADA (*n* = 57)	*P*-value
**Patients with ≥1 severe adverse events, *n* (%)**	16 (13%)	2 (4%)	0.082
**Number of hospitalizations^a^**			
** Luminal surgery**	6	0	
** Disease worsening**	10	2	
** Treatment related complication^b^**	2	0	
** Peri-anal abscess drainage**	1	0	

Abbreviations: ADA; adalimumab; IFX, infliximab.

a Patients could have multiple hospitalizations within 1 year.

b Treatment related complications included infusion reaction (*n* = 1) and azathioprine induced pancreatitis (*n* = 1).

#### Treatment duration

**Figure 2:**
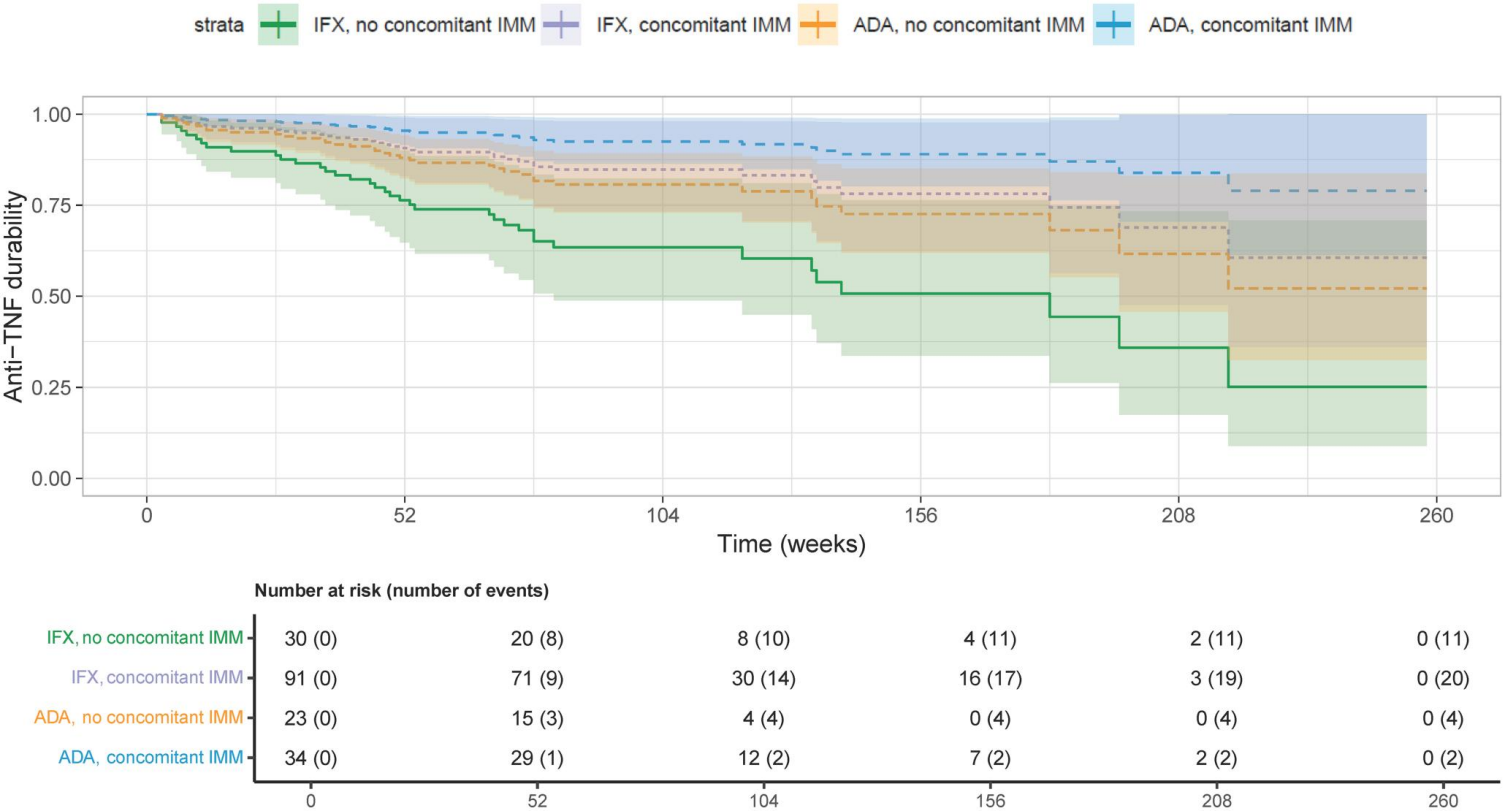
Survival curve showing the estimated probability of anti-TNF durability stratified by anti-TNF type and concomitant immunomodulator use. ADA, adalimumab; IFX, infliximab; IMM, immunomodulatory; TNF, tumor necrosis factor.

Median follow-up duration from start of anti-TNF treatment until the last recorded visit was 101 weeks (IQR 63-158) for infliximab and 94 weeks (IQR 59-112) for adalimumab. Until last follow-up, 6/57 patients (11%) treated with adalimumab discontinued their treatment (4/57 [7%] within the first year) compared to 26/121 patients (21%) treated with infliximab (14/121 [12%] within the first year), excluding 5/121 (4%) of patients who stopped due to sustained remission. None of the patients treated with adalimumab underwent luminal surgery until last follow-up, 8/121 (7%) patients treated with infliximab underwent luminal surgery until last follow-up (6/121 [5%] within the first year). The 1-year unadjusted survival probability for infliximab was 0.86 (95% CI, 0.80-0.92) and for adalimumab 0.93 (95% CI 0.86-1.00). In both groups, LOR was the most frequent reason for anti-TNF discontinuation (infliximab *n* = 8 [7%], adalimumab *n* = 3 [5%]) (Table 3). Adalimumab was associated with a significant lower adjusted hazard ratio (aHR) of treatment discontinuation than infliximab in patients with a concomitant immunomodulator (aHR 0.17 [95% CI, 0.04-0.75], *P* = .020) but not in patients without a concomitant immunomodulator at start of anti-TNF treatment (aHR 0.53 [95% CI, 0.17-1.70], *P* = .288) (Figure 2). Our study did not observe an effect of concomitant immunomodulator on the durability of anti-TNF treatment after adjusting for the timing of anti-TNF administration (*P-*value of overall effect = 0.08), for adalimumab (aHR 0.21 [95% CI, 0.04-1.13, *P* = .070]) and infliximab (aHR 0.63 [95% CI, 0.30-1.33], *P* = .225). Upfront anti-TNF treatment was associated with a decrease in the hazard of discontinuation of anti-TNF treatment (aHR 0.38 [95% CI, 0.17-0.85], *P* = .019). Survival probabilities stratified by treatment, timing of anti-TNF initiation and concomitant immunomodulator are shown in Table S3.

**Table 3:** Reasons for discontinuation of anti-TNF treatment in children with Crohn’s disease.

	IFX (*n* = 121)	ADA (*n* = 57)
**Patients with anti-TNF discontinuation, *n* (%)**	31 (26)	6 (11)
**Reason for discontinuation, *n* (%)**		
** Primary non-response**	4 (3)	0
** Loss of response**	8 (7)	3 (5)
** Development of antibodies^a^**	6 (5)	0
** Side-effects**	3 (2)	2 (4)
** Sustained remission**	5 (4)	0
** Other or multiple reasons^b^**	3 (2)	1 (2)
** Unknown**	2 (2)	0

Abbreviations: ADA; adalimumab; IFX, infliximab; TNF, tumor necrosis factor.

a Four out of six patients who developed anti-drug antibodies received a concomitant immunomodulator (defined as initation before or within 2 weeks of starting anti-TNF therapy, and was given for at least 1 week after start of anti-TNF).

b Other reasons for discontinuation for IFX were severe anxiety for IFX infusion (*n* = 1), very painful infusions (*n* = 1), or noncompliance (*n* = 2; 1 infliximab and 1 adalimumab).

## Discussion

In this large, prospective, international, pediatric cohort study, no significant differences were found in steroid free clinical remission outcomes or rates of SAEs at 12 months between adalimumab and infliximab in biologic-naïve children with CD. Moreover, we found a significant difference in the treatment durability in favor of adalimumab for patients who were on a concomitant immunomodulator at start of anti-TNF treatment, but this effect was not observed in the minority of patients who were not on a concomitant immunomodulator.

Previous adult and pediatric studies have generally not shown significant differences in effectiveness between infliximab and adalimumab.^7–14^ For example, DeBruyn et al. compared infliximab and adalimumab treatment in a nationwide Canadian observational cohort of children with CD. Based on a propensity-score matched cohort of 294 patients, no significant difference was found in SFCR at 1 year of adalimumab compared to infliximab (OR 1.4 [95% CI, 0.9-2.4]).^7^ An Italian registry of pediatric patients with CD likewise showed that infliximab had lower durability compared to adalimumab (HR 2.0 [95% CI, 1.5-2.7], *P* < 0.001).^15^

Although we found no significant differences in SFCR, SFMI, and NCR, adalimumab combotherapy was associated with a lower adjusted HR of treatment discontinuation compared to infliximab combotherapy, although this finding was not corrected for disease severity at anti-TNF commencement In contrast, deBruyn et al. found no significant difference in treatment discontinuation between both therapies and reported slightly higher discontinuation rates in adalimumab-treated patients after 1 year (12%) than in our study (7%).^7^ These higher discontinuation rates for adalimumab in their study may be due to the fact that in the propensity score matched cohort of deBruyn et al., despite comparable rates of concomitant immunomodulator, patients starting adalimumab had a longer time to anti-TNF initiation and higher disease activity at baseline than in our study. Indeed, our results indicated that upfront anti-TNF therapy was associated with improved treatment durability. Starting effective therapy as soon as possible is important to reduce the risk of complications.^27^ Previous studies have shown that early anti-TNF therapy is more effective compared to late initiation in children with CD.^28^^,^^29^

One reason for the longer durability of adalimumab-treated patients compared to infliximab-treated patients observed in our cohort may be the lower proportion of patients developing antibodies (0%) compared to infliximab (5%). This could be due to the nature of the 2 treatments, as infliximab is a partially chimeric antibody, while adalimumab is fully humanized. Additionally, adalimumab is administered subcutaneously with a lower dose interval than infliximab, resulting in more consistent and less suboptimal trough levels, which may be associated with a lower likelihood of developing antibodies.^30^^,^^31^ The prospective multicenter Personalized ANti-TNF Therapy in Crohn’s disease Study (PANTS), recently published by Chanchlani et al., similarly showed higher rates of antibody formation in infliximab-treated patients compared to those treated with adalimumab.^31^

To reduce immunogenicity and ensure adequate drug levels improving treatment durability, the current ECCO-ESPGHAN guideline recommends administering a concomitant immunomodulator when initiating infliximab treatment, and to consider this for adalimumab.^1^ This probably explains why rates of combination treatment with an immunomodulator were somewhat higher for infliximab in our study. Interestingly, our study did not find an overall or modifying effect of concomitant immunomodulator on anti-TNF durability when adjusting for timing of anti-TNF. However, given the low number of events of treatment discontinuation we might have been underpowered and were not able to correct for other possible confounders such as disease activity at baseline.

There is conflicting evidence on whether adding an immunomodulator to infliximab or adalimumab indeed leads to improved outcomes. One study including 66 children with CD treated with infliximab monotherapy with proactive therapeutic drug monitoring (54% of patients with a trough level <5 μg/mL at the fourth infusion received infliximab dose intensification) and 62 children with CD treated with infliximab combination therapy found no differences in clearance or trough levels after 5 infusions.^33^ On the other hand, the PANTS study demonstrated that concomitant immunomodulator use was associated with lower antibody development with undetectable drug concentrations for both infliximab and adalimumab, but with no difference in effect size between both therapies (HR for infliximab 0.40 [95% CI, 0.31-0.52] and HR for adalimumab 0.42 [95% CI, 0.24-0.75]).^32^ Similarly, in a population-based Israelian study, Atia et al. showed improved but comparable treatment durability with combination therapy in both infliximab and adalimumab. Adalimumab monotherapy was found to have higher durability than infliximab monotherapy, but not higher than infliximab combination therapy.^34^ A randomized controlled trial in children with CD which compared anti-TNF monotherapy to combination therapy (with low-dose methotrexate) found that infliximab combination therapy was not associated with lower rates of treatment discontinuation compared to infliximab monotherapy (HR 0.93 [95% CI, 0.55-1.56]), but that adalimumab combination therapy was associated with lower rates of treatment failure in adalimumab treated patients (HR 0.30, 95% CI, 0.19-0.51).^35^ In summary, most studies seem to support combination therapy, but further studies are required, particularly studies in which pro-active therapeutic drug monitoring is applied.

In addition to the clinical effectiveness and durability of the 2 treatments, other factors should be considered when prescribing either infliximab or adalimumab. These factors include adverse events, adherence, and patient and physician preferences. We found no significant difference in adverse events between the 2 groups, which aligns with previous findings.^7^^,^^12^ Monitoring therapy adherence is easier when patients are required to visit the hospital for an infusion, as opposed to administering subcutaneous medication at home, which might be a reason for non-adherence. It should be noted that subcutaneous administration of infliximab is becoming more common, and data in adults have shown promising results.^36–38^ In children, published data is limited with results available only from a small sample of 7 patients with CD.^39^ Another factor that could be considered is the necessity to prescribe a concomitant immunomodulator with infliximab and/or adalimumab. Although the ECCO-ESPGHAN guideline recommends that adalimumab may be prescribed as monotherapy and infliximab as a combination therapy, as stated above, there is conflicting evidence whether a concomitant immunomodulator is (only) beneficial for infliximab. Combination therapy has certain disadvantages, for example, although rare, azathioprine may lead to some rare but serious side effects such as bone marrow suppression, pancreatitis or hepatosplenic T-cell lymphoma, and methotrexate frequently results in side effects such as nausea and may be inconvenient for the patient, making monotherapy a preferable treatment approach. Furthermore, pediatric gastroenterologists tend to prescribe infliximab more often to patients with a higher inflammatory burden or those who have perianal disease, due to a more rapid onset of action and higher drug levels during induction with intravenous infusion of infliximab. However, comparative data of both anti-TNF therapies in these specific subgroups is scarce. In the study of DeBruyn et al., before matching, patients receiving infliximab were more likely to have perianal disease or severe disease.^7^ The results of our study also showed that patients with infliximab had higher disease activity, indicated by higher clinical disease activity, levels of CRP and ESR and lower albumin levels. Additionally, among patients treated with infliximab, there was a numerically higher proportion of patients with perianal disease. In the study by DeBruyn et al., outcomes at 1 year were compared in the moderate-to-severe subgroup, showing no differences between infliximab and adalimumab. In pediatric patients with an acute severe (ulcerative) colitis, guidelines recommend treating with infliximab rather than adalimumab as rescue therapy after failing intravenous corticosteroids.^40^ Outcomes after induction have also been better with infliximab in adult patients with moderate-to-severe UC, but long-term outcomes are generally comparable.^9^^,^^41–44^ For patients with perianal disease, current evidence is insufficient to clearly favor infliximab over adalimumab,^45^ as supported by the pediatric CD guideline.^1^ Unfortunately, the sample size in our study was too small to perform a comparison of adalimumab and infliximab in subgroups of patients with peri-anal disease or moderate-to-severe disease. Further studies are required to investigate the effectiveness of these drugs in these subgroups.

A major strength of this study is that extensive clinical data were prospectively collected in an international cohort, increasing the generalizability and reducing the risk of recall bias. Another strength of this study is that we used the method of standardization, reducing the risk of confounding by indication. However, this study also has limitations. As this study is not an RCT, we could not account for potential unmeasured confounders, which might have introduced bias. Additionally, although data was collected prospectively, bias may have been introduced due to retrospective nature of the analysis. Furthermore, the presence of missing data required imputation, and the use of the PGA score for imputing wPCDAI scores may also have influenced the results. Follow-up was relatively short to study treatment durability, and the low number of events did not allow us to include sufficient confounders (such as disease severity at baseline) in our Cox PH model. These results should thus be interpreted with caution. Due to the small number of patients with perianal disease, we were unable to assess differences in clinical effectiveness of infliximab and adalimumab specifically in patients with perianal disease. Another limitation of this study is that, due to the study design and selection of the 1-year visit, patients who began treatment within the first weeks after diagnosis were more likely to have a 1-year visit and thus less likely to be excluded. This is why there was a relatively high proportion of patients receiving early anti-TNF therapy. Another limitation was the absence of routinely collected data on fecal calprotectin and trough levels for both treatments. Furthermore, we did not take into account endoscopic or radiology outcomes after start of anti-TNF, (changes in) dosing regimens of anti-TNF treatment, differences in treatment strategy between centers/countries, adherence to treatment, nor did we evaluate the impact of drug type (biosimilar or originator) or formula (eg, citrate-free injections). Last, SAEs and side effects might have been inconsistently reported.

In conclusion, there were no significant differences in clinical effectiveness between infliximab and adalimumab at 1 year. Both anti-TNF therapies are effective treatments options in children with CD, and the decision should be made based on patient and physician preferences. Further research should focus on comparing subcutaneous infliximab with subcutaneous adalimumab, as well as investigating whether adding a concomitant immunomodulator improves clinical outcomes and durability for any anti-TNF.

## Supplementary Material

otag069_Supplementary_Data

## Data Availability

The data underlying this article will be shared on reasonable request to the corresponding author.
