# Supplementary material for: Comparative effectiveness of adalimumab versus infliximab in children with Crohn’s disease: real-world data from the prospective PIBD-SETQuality inception cohort study
Source: Crohns Colitis 360. 2026 Jul 3;8(3):otag069. doi: 10.1093/crocol/otag069 (PMC13431788; doi:10.1093/crocol/otag069)
Supplement: otag069_Supplementary_Data [file otag069_supplementary_data.docx]

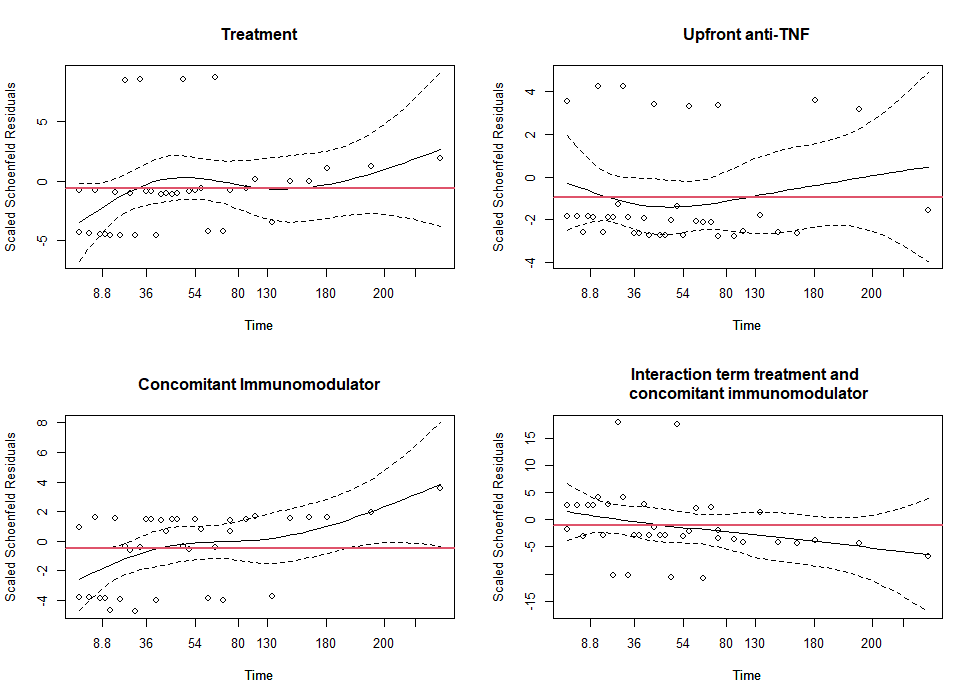
**SUPPLEMENTARY MATERIAL

Supplementary Figure 1. Plot of Schoenfeld residuals for the evaluation of the proportional hazard assumption per included variable.**

TNF; tumor necrosis factor

**Supplementary Table 1. Disease activity at baseline and at 12 months when comparing patients with and without missing fecal calprotectin values at 12 months.**

|  | Patients without missing fecal calprotectin at 12 months (n=86) | Patients with missing fecal calprotectin at 12 months (n=92) | P-value | |
| --- | --- | --- | --- | --- |
| Disease activity at baseline  Remission  Mild  Moderate  Severe | | 18 (21%)  22 (26%)  24 (28%)  22 (26%) | 19 (21%)  40 (43%)  15 (16%)  18 (20%) | 0.057 |
| Disease activity at 12 months  Remission  Mild  Moderate  Severe | | 63 (74%)  21 (25%)  1 (1%)  0 | 58 (64%)  27 (30%)  4 (4%)  1 (1%) | 0.260 |

Disease activity at 12 months is missing in 3 patients.

**Supplementary Table 2. Side effects within 12 months after start of anti-TNF treatment in children with Crohn’s disease.**

|  | IFX (n = 121) | ADA (n = 57) | p-value |
| --- | --- | --- | --- |
| Patients with ≥1 side effects, n (%) | 30 (25%) | 7 (12%) | 0.09 |
| Reported side effects, n  Abdominal pain  Headache  Infection  Opportunistic infection  Arthritis  Infusion reaction  Psoriasiform skin eruption  Other  Dry skin  (Exacerbation) eczema  Skin rash  Nausea  Fatigue  Other | 4  8  1  0  2  6  1  25  3  5  2  3  5  7 | 1  0  1  1  0  2  2  3  0  0  1  0  1  1 |  |

Patients could have had multiple side effects. Other reported side effects for patients treated with IFX were pityriasis capitis n=1, sweating n=1, night sweats n=1, dyspnea n=1, chest pain n=1, enlarged lymph node n=1, moodswings n=1 and for patients treated with ADA cervical neck pain n=1. Patients could have multiple side effects within 1 year. IFX; infliximab, ADA; adalimumab

**Supplementary Table 3. Survival probabilities at 1, 2, and 3 years after start of anti-TNF treatment in children with Crohn’s disease.**

|  | 1 year | 2 years | 3 years |
| --- | --- | --- | --- |
| Anti-TNF type: infliximab |  |  |  |
| No upfront anti-TNF  No concomitant IMM | 0.74 (95% CI 0.60 - 0.91) | 0.59 (95% CI 0.42 - 0.84) | 0.30 (95% CI 0.12 - 0.73) |
| No upfront anti-TNF  Concomitant IMM | 0.83 (95% CI 0.74 - 0.92) | 0.72 (95% CI 0.61 - 0.86) | 0.47 (95% CI 0.28 - 0.79) |
| Upfront anti-TNF  No concomitant IMM | 0.89 (95% CI 0.79 – 0.99) | 0.82 (95% CI 0.68 – 0.99) | 0.63 (95% CI 0.40 – 0.99) |
| Upfront anti-TNF  Concomitant IMM | 0.93 (95% CI 0.88 - 0.98) | 0.88 (95% CI 0.80 - 0.97) | 0.75 (95% CI 0.58 - 0.96) |
| Anti-TNF type: adalimumab |  |  |  |
| No upfront anti-TNF  No concomitant IMM | 0.85 (95% CI 0.72 – 1.00) | 0.76 (95% CI 0.57 – 1.00) | 0.53 (95% CI 0.26 – 1.00) |
| No upfront anti-TNF  Concomitant IMM | 0.97 (95% CI 0.92 – 1.00) | 0.94 (95% CI 0.87 – 1.00) | 0.88 (95% CI 0.72 – 1.00) |
| Upfront anti-TNF  No concomitant IMM | 0.94 (95% CI 0.87 – 1.00) | 0.90 (95% CI 0.79 – 1.00) | 0.78 (95% CI 0.57 – 1.00) |
| Upfront anti-TNF  Concomitant IMM | 0.99 (95% CI 0.97 - 1.00) | 0.98 (95% CI 0.94 – 1.00) | 0.95 (95% CI 0.88 -1.00) |

TNF: tumor necrosis factor, IMM: immunomodulator

**R-packages**

The Mann-Whitney U test, Chi-squared and Fisher exact test were calculated using the R-package stats (version 4.3.1, R Core Team, 2023). Standardization was done using the R-packages arm (version 1.14.4, Gelman and Su, 2024) and boot (version 1.3.30, Canty and Ripley, 2024). For imputation, the following packages were used: mitools (version 2.4, Lumley, 2019), mice (version 3.16.0, van Buuren and Groothuis-Oudshoorn, 2011), miceadds (version 3.17.44, Robitzsch and Grund, 2024). (Penalized) logistic regression was done using R-package lme4 (version 1.1.35.3, Bates, Macheler, Bolker, Walker, 2015). Survival analyses were performed using R-package survival (version 3.5.5., Therneau, 2023). Data was formatted by R-packages dplyr (version 1.1.4, Wickham, François, Henry, Müller, Vaughan, 2023). Figures were made using R-package ggplot2 (version 3.5.1, Wickham, 2016) and survminer (version 0.4.9, Kassambara, Kosinski, Biecek, 2021).
